# Subpathway Analysis based on Signaling-Pathway Impact Analysis of Signaling Pathway

**DOI:** 10.1371/journal.pone.0132813

**Published:** 2015-07-24

**Authors:** Xianbin Li, Liangzhong Shen, Xuequn Shang, Wenbin Liu

**Affiliations:** 1 Department of Physics and Electronic information engineering, Wenzhou University,Wenzhou, Zhejiang, China; 2 School of Computer Science and Technology, Northwestern Polytechnical University, Xi'an, China; Centro de Investigacion Principe Felipe, SPAIN

## Abstract

Pathway analysis is a common approach to gain insight from biological experiments. Signaling-pathway impact analysis (SPIA) is one such method and combines both the classical enrichment analysis and the actual perturbation on a given pathway. Because this method focuses on a single pathway, its resolution generally is not very high because the differentially expressed genes may be enriched in a local region of the pathway. In the present work, to identify cancer-related pathways, we incorporated a recent subpathway analysis method into the SPIA method to form the “sub-SPIA method.” The original subpathway analysis uses the k-clique structure to define a subpathway. However, it is not sufficiently flexible to capture subpathways with complex structure and usually results in many overlapping subpathways. We therefore propose using the minimal-spanning-tree structure to find a subpathway. We apply this approach to colorectal cancer and lung cancer datasets, and our results show that sub-SPIA can identify many significant pathways associated with each specific cancer that other methods miss. Based on the entire pathway network in the Kyoto Encyclopedia of Genes and Genomes, we find that the pathways identified by sub-SPIA not only have the largest average degree, but also are more closely connected than those identified by other methods. This result suggests that the abnormality signal propagating through them might be responsible for the specific cancer or disease.

## Introduction

Various “omics” technologies, such as microarrays, RNAseq, and gas chromatography mass spectrometry, can help to identify potentially interesting (i.e., differential) genes and metabolites, especially those associated with specific diseases. However, using such information to better understand the underlying biological phenomena remains a challenge. Pathway analysis has become a popular approach for gaining insight into the underlying biology of differentially expressed genes (DEGs) and proteins. The evolution of knowledge-driven pathway analysis can be divided into four generations. The first-generation analysis method is called the overrepresentation approach (ORA) [1] and compares the number of differential genes expected to hit the given pathway by chance. If this number differs significantly from that expected by chance, the pathway is significant. Many tools are based on first-generation methods, such as Onto-Express [2, 3] or GOEASE [4]. The ORA assumes that each gene is independent of the other genes. However, biological processes form a complex web of interactions between gene products that constitute different pathways. Functional class scoring (FCS) is a second-generation method for detecting coordinated changes in the expression of genes in the same pathway. Gene-set enrichment analysis is an example of a second-generation method [5, 6]. Because upstream genes may have a larger impact than downstream genes, pathway-topology-based approaches, such as signaling-pathway impact analysis (SPIA) [7] and ScorePAGE [8], qualify as third-generation methods. In particular, SPIA combines classical enrichment analysis and the perturbation on a given pathway, which allows it to capture the influence of upstream genes. Following SPIA, Vaske *et al*. [9] proposed a method named PARADIGM, which integrates diverse high-throughput genomics information with known signaling pathways to provide patient-specific genomic inferences on the state of gene activities, complexes, and cellular processes. Recently, researchers proposed that key subpathway regions may represent the corresponding pathway and be more relevant for interpreting the associated biological phenomena. Moreover, several studies show that abnormalities in subpathway regions of metabolic pathways may contribute to the etiology of diseases [10–12]. Subpathway analysis in signaling pathways has also been studied, resulting in approaches such as DEgraph [13], the clipper approach [14], and Pathiways [15]. These are qualified as fourth-generation methods.

In the present work, we combine the approaches of subpathway analysis and SPIA, which we call sub-SPIA, to identify biologically meaningful signaling pathways. One key problem in subpathway analysis is how to define a subpathway. Li *et al*. [11] used the k-clique concept to define a subpathway. However, pathways are usually sparsely connected and are composed of many linear structures. The k-clique concept has two limitations: (i) the relationship between DEGs in a subpathway may not exactly form a k-clique structure, and (ii) the k-clique algorithm usually results in many redundant and overlapped subpathways. The minimal-spanning tree (MST) is a simple data structure that is frequently used to represent tightly related nodes in a graph. It is more appropriate to represent various subpathways than the k-clique structure, especially in sparse-pathway networks.

We applied the sub-SPIA method to the colorectal cancer (CRC) and lung cancer datasets, and our results demonstrate that the proposed method can identify more disease-related pathways than SPIA, DEgraph, Clipper, and Pathiways. Furthermore, we find that most of the pathways identified by sub-SPIA have a high degree and are tightly connected within the entire pathway network. This result reveals that diseases (e.g., cancer) may result from the synergic interactions of a group of related pathways.

## Results

There are 137 signaling pathways in KEGG. To deduce pathway significance, we used a significance threshold of 1% on the p-values corrected for false discovery rate (FDR). For both sub-SPIA and SPIA, the FDR-adjusted p-values *P*_*G*_(FDR) which combine the enrichment and perturbation p-values (see definition in the method section), were computed from the nominal p-values by using the *R* function “p.adjust.” We present herein the significantly enriched pathways by applying sub-SPIA and SPIA to CRC and lung cancer datasets. The significant pathways that were also identified by DEgraph, Clipper, and Pathiways and are given in Tables 1 and 2.

**Table 1:** Significantly enriched pathways identified by sub-SPIA and SPIA from the CRC dataset.

No	Pathway	Sub-SPIA	SPIA	Clipper	Kclique	Pathiways	DEGraph	Ref
**1**	Focal adhesion	1.30E-10	1.72E-08		Yes			[16, 17]
**2**	PPAR signaling pathway	1.30E-10	4.67E-05		Yes	Yes		
**3**	ECM-receptor interaction	7.90E-07	7.19E-06		Yes	Yes		
**4**	Pathways in cancer	0.0001	0.0011					
**5**	Regulation of actin cytoskeleton	1.88E-06	0.071		Yes			[18, 19]
**6**	MAPK signaling pathway	2.81E-06	0.056		Yes			[20–27]
**7**	Complement and coagulation cascades	2.76E-05	0.79					[35, 36]
**8**	Wnt signaling pathway	0.0007	0.066		Yes			[28–32]
**9**	Staphylococcus aureus infection	0.0018	0.26					[40]
**10**	p53 signaling pathway	0.0019	0.7429			Yes		[33, 34]
**11**	Notch signaling pathway	0.0029	0.2185		Yes			[39,75,76]
**12**	Renal cell carcinoma	0.0037	0.0800				Yes	[77, 78]
**13**	ErbB signaling pathway	0.0037	0.2207		Yes			[42–45]
**14**	T cell receptor signaling pathway	0.0045	0.4230			Yes		[41]
**15**	Circadian rhythm	0.0045	0.1659					
**16**	Tuberculosis	0.0045	0.3576					[49]
**17**	Dopaminergic synapse	0.0046	0.2185					[79]
**18**	Legionellosis	0.0046	0.1926		Yes		Yes	
**19**	Axon guidance	0.0104	0.0002					[80–82]
**20**	Parkinson's disease	0.0439	1.19E-09					
**21**	Alzheimer's disease	0.1827	1.19E-09					
**22**	Huntington's disease	No	4.67E-05					

**Table 2:** Significantly enriched pathways identified by sub-SPIA and SPIA from lung cancer dataset.

No	Pathway	Sub-SPIA	SPIA	Clipper	Kclique	Pathiways	DEGraph	Ref
**1**	ECM-receptor interaction	3.17E-05	7.60E-05	Yes				
**2**	Cell cycle	3.17E-05	0.0652	Yes	Yes			[83]
**3**	Focal adhesion	3.17E-05	0.0109					[51, 52]
**4**	Tuberculosis	0.0002	0.8017				Yes	[65]
**5**	NF-kappa B signaling pathway	0.0008	0.2694	Yes				[62, 84]
**6**	p53 signaling pathway	0.0010	0.3529	Yes				[85, 86]
**7**	Melanogenesis	0.0010	0.580	Yes				
**8**	PPAR signaling pathway	0.0010	0.5399	Yes			Yes	
**9**	MAPK signaling pathway	0.0016	0.1993				Yes	[54]
**10**	Fc gamma R-mediated phagocytosis	0.0016	0.0595	Yes			Yes	
**11**	Regulation of actin cytoskeleton	0.0019	0.0581					[53]
**12**	Pathways in cancer	0.0020	0.0581					
**13**	Fanconi anemia pathway	0.0023	0.0651					[63]
**14**	Wnt signaling pathway	0.0025	0.0581	Yes				[55]
**15**	Amphetamine addiction	0.0046	0.8161	Yes			Yes	
**16**	Vascular smooth muscle contraction	0.0057	0.0652	Yes				[87]
**17**	Salmonella infection	0.0068	0.1034	Yes	Yes		Yes	[88]
**18**	Complement and coagulation cascades	0.0068	0.4547	Yes				
**19**	Staphylococcus aureus infection	0.0088	0.3529	Yes				
**20**	Protein processing in endoplasmic reticulum	0.1649	7.60E-05		Yes			
**21**	RNA transport	0.2624	0.0006					
**22**	Epstein-Barr virus infection	0.4085	0.0053	Yes			Yes	[69]
**23**	Bacterial invasion of epithelial cells	0.0797	0.0087	Yes	Yes			

### Pathway Analysis

#### Colorectal-Cancer Dataset

Sub-SPIA identified 18 potential pathways associated with CRC, while SPIA identified only 8 pathways. These pathways and their corresponding *P*_*G*_ are listed in Table 1. Four common pathways were identified by both SPIA and sub-SPIA: *focal adhesion*, *pathways in cancer*, *PPAR signaling pathway*, *and ECM-receptor interaction*. Previous studies show that some common pathways are highly associated with various cancers, including CRC and lung cancer, such as *focal adhesion [16, 17], pathways in cancer, regulation of actin cytoskeleton [18, 19], the MAPK signaling pathway [20–27], ECM-receptor interaction, the Wnt signaling pathway [28–32],* and the *p53 signaling pathway [33, 34]*. Sub-SPIA identified all of these pathways, whereas SPIA only identified three of them. However, some pathways are not likely to be relevant to CRC, such as the pathways for *Huntington’s disease*, *Alzheimer's disease*, and *Parkinson’s disease* [7]. These are ranked first, second, and sixth in significance by SPIA, whereas sub-SPIA identifies them either as not significant or not found.

Concerning the significant pathways identified by sub-SPIA but not by SPIA, existing evidence indicates that they may be associated with CRC. The dysregulation of the *regulation of actin cytoskeleton* pathway plays a key role in the progression of CRC [18, 19]; this pathway contains a differential gene LIMK2 that promotes tumor-cell invasion and metastasis. LIMK2 expression is associated with CRC progression, and its deletion affects gastrointestinal stem cell regulation and tumor development.

The *complement and coagulation cascades pathway* involves 14 DEGs and also plays a key role in the progression of CRC. For example, in a comparison of radiosensitive and radio-resistant lines of CRC cells, the result of five lines of CRC cells shows that 30 up-regulated genes were identified as being involved in *DNA damage response* pathways, *immune response* pathways, and the *complement and coagulation cascades* pathway [35, 36].

**Fig 1:**
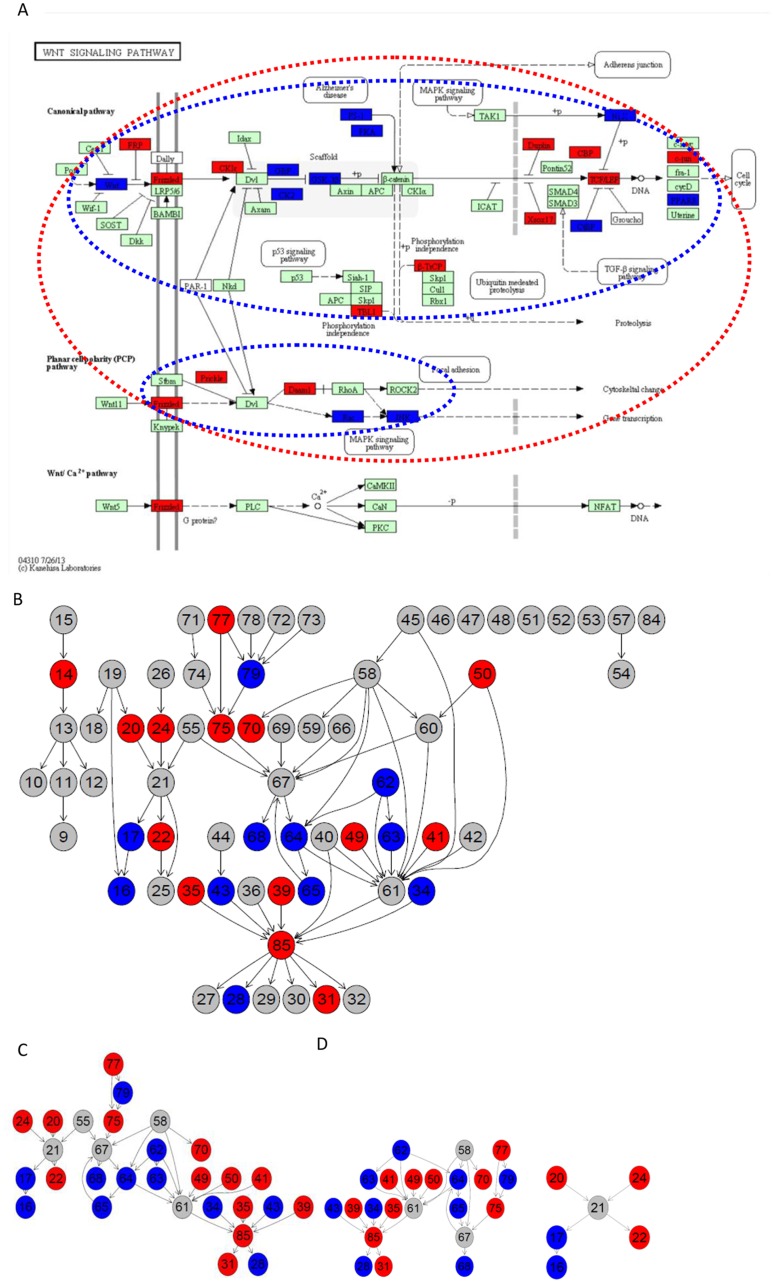
Subpathways in the Wnt signaling pathway identified by Sub-SPIA on CRC dataset. (A) The insulin-signal pathway with DEGs highlighted. (B) Gene network obtained by graphite package. (C) Sub Gene network corresponding to the MST for Minimal-spanning tree for *n_s_* = 4. (D) Sub Gene networks corresponding to the MST for Minimal-spanning tree for *n_s_* = 2. Red indicates an up-regulated gene and blue indicates a down-regulated gene. Reprinted from http://www.kegg.jp/kegg/kegg1.html under a CC BY license, with permission from Miwako Karikomi, original copyright 2013.

The deregulation of the *notch signaling pathway* was observed in colorectal and other forms of cancer [37]. NOTCH is the center of the subpathway identified by sub-SPIA (see Fig 1). The abnormal expression of NOTCH and its upstream gene NUMB would lead to the dysfunction of many downstream genes. The Notch signaling pathway is involved in regulating stem-cell hierarchy and determining cell fate [38]. A recent study [39] indicates that inhibiting prolactin can completely abrogate the Notch signaling pathway and may provide a novel target for therapeutic intervention.

Elafin is a protease inhibitor with antibacterial effects against bacteria such as *Pseudomonas aeruginosa* and *Staphylococcus aureus*, and which modulates inflammation through its antiprotease activity. A delicate relationship between proteases and antiproteases is central in determining how inflammation develops during colitis. Proteases damage tissues during inflammation whereas protease inhibitors minimize tissue damage and facilitate healing. In a microarray study of human colonic biopsies, patients with ulcerative colitis expressed 30-fold more elafin mRNA than healthy controls, which indirectly indicates that the *Staphylococcus aureus infection* pathway is associated with CRC [40].

The *T cell receptor signaling* pathway is responsible for impaired immune responsiveness of T cells in cancer patients. In such patients, the TCR-β gene in lymphocytes is less expressed in colon cancer, renal cell carcinoma, melanoma, and cervical cancer, which has important clinical implications for monitoring the patient immune status during therapy [41].

The human epidermal growth factor receptor family contains four members that belong to the ErbB lineage of proteins (ErbB1-4)[42–45] [42–45]. Downstream *ErbB signaling* modules include the phosphatidylinositol 3-kinase/Akt pathway, the Ras/Raf/MEK/ERK1/2 pathway, and the phospholipase C pathway. Several malignancies are associated with the mutation or increased expression of members of the ErbB family, including lung, breast, and stomach cancer [42]. By immunohistochemistry, Yao *et al*. found that receptor tyrosine kinase (RTK) members ErbB2, ErbB3, and c-Met were indeed differentially overexpressed in samples from CRC patients, leading to constitutive activation of RTK signaling pathways. By using the ErbB2-specific inhibitor Lapatinib and the c-Met-specific inhibitor PHA-665752, they further demonstrated that this constitutive activation of RTK signaling is necessary to the survival of CRC cells [46].

As of 2008, CRC is the third most commonly diagnosed cancer in males and the second in females [47]. Worldwide, *mycobacterium tuberculosis* (MTB) is the second leading cause of death from an infectious disease [48]. Significant evidence shows that active MTB could be present in patients with metastatic CRC [49]. Although the samples we used were not reported to be infected with these pathogens, it is possible that the *tuberculosis* pathway plays an important role in them and thus is identified by sub-SPIA as being significant in CRC.

### Lung Cancer Dataset

Sub-SPIA identified 19 potential pathways associated with lung cancer, whereas SPIA only identified 5 pathways (see S2 Table). These pathways and their corresponding *P*_*G*_ are listed in Table 2. Both methods identified three common pathways: *ECM-receptor interaction*, *cell cycle*, and *focal adhesion*. Qiu *et al.[50]* found that DEGs may promote metastasis of lung cancer cells through complicated networks, including pathways in *cancer, focal adhesion [51, 52], regulation of actin cytoskeleton [53], the p53 signaling pathway, the MAPK signaling pathway [54], ECM-receptor interaction, and the Wnt signaling pathway [55]*. These pathways are also identified by sub-SPIA, whereas SPIA only identifies five of them with *P*<0.06.

Gene anomalies in *cell-cycle* pathways have been frequently observed in a variety of human malignancies, including lung cancer [56–58]. Dysfunctions of proto-oncogenes, such as *CCND1* and *STK15*, and tumor-suppressor genes, such as *p53*, *p21*, and *p27*, are commonly associated with increased cell proliferation, defective apoptosis, elevated cancer risk, and poor survival rates [50, 59].

Constitutive activation of NF-κB was detected in non-small-cell lung carcinoma (NSCLC) and was implicated in imparting resistance to CDDP [60, 61]. Therefore, inhibiting *NF-κB* signaling may be a critical target for enhancing the efficacy of CDDP against NSCLC. Wang *et al*. [62] found that the inhibition of NF-κB by geldanamycin (GA) could be responsible for the synergistic apoptosis-inducing effect of GA and CDDP in NSCLC cells and tumor xenografts.

By using methylation-specific PCR, Marsit *et al*. [63] found the epigenetic alterations in the *fanconi anemia* pathway in NSCLC. They demonstrated that inactivation of the FANC-BRCA pathway is relatively common in solid tumors and may be related to tobacco and alcohol exposure and to the survival of these patients.

Aberrant activities of the *vascular smooth muscle contraction* and the *focal adhesion* pathways may play key roles in the initiation and development of NSCLC. Fang *et al*. [64] demonstrated the indispensable roles of these two signal pathways in the carcinogenesis of NSCLC.

The comorbidity of lung cancer and pulmonary *tuberculosis* (TB) is a clinical problem whose diagnosis and treatment presents a challenge [65]. In a review of 36 patients with *salmonella* pneumonia, or lung abscess, Cohen *et al*. noted that thirteen of them (36%) had prior abnormalities of the lung or pleura. From among these thirteen, seven had lung malignancies [66]. Although the samples we used were not reported to be infected with *salmonella* pathogens, it is possible that the *tuberculosis* pathway plays an important role in them and thus is identified by sub-SPIA.

### Subpathway Analysis

According to the KEGG pathway hsa05200 (also called pathways in cancer), the signals from the outside pathways, such as *Wnt* signaling and *MAPK* signaling, are common driving forces during carcinogenesis [15]. The sub-SPIA not only improves the identification resolution of cancer-related pathways, but also helps biologists to understand their underlying mechanisms.

**Fig 2:**
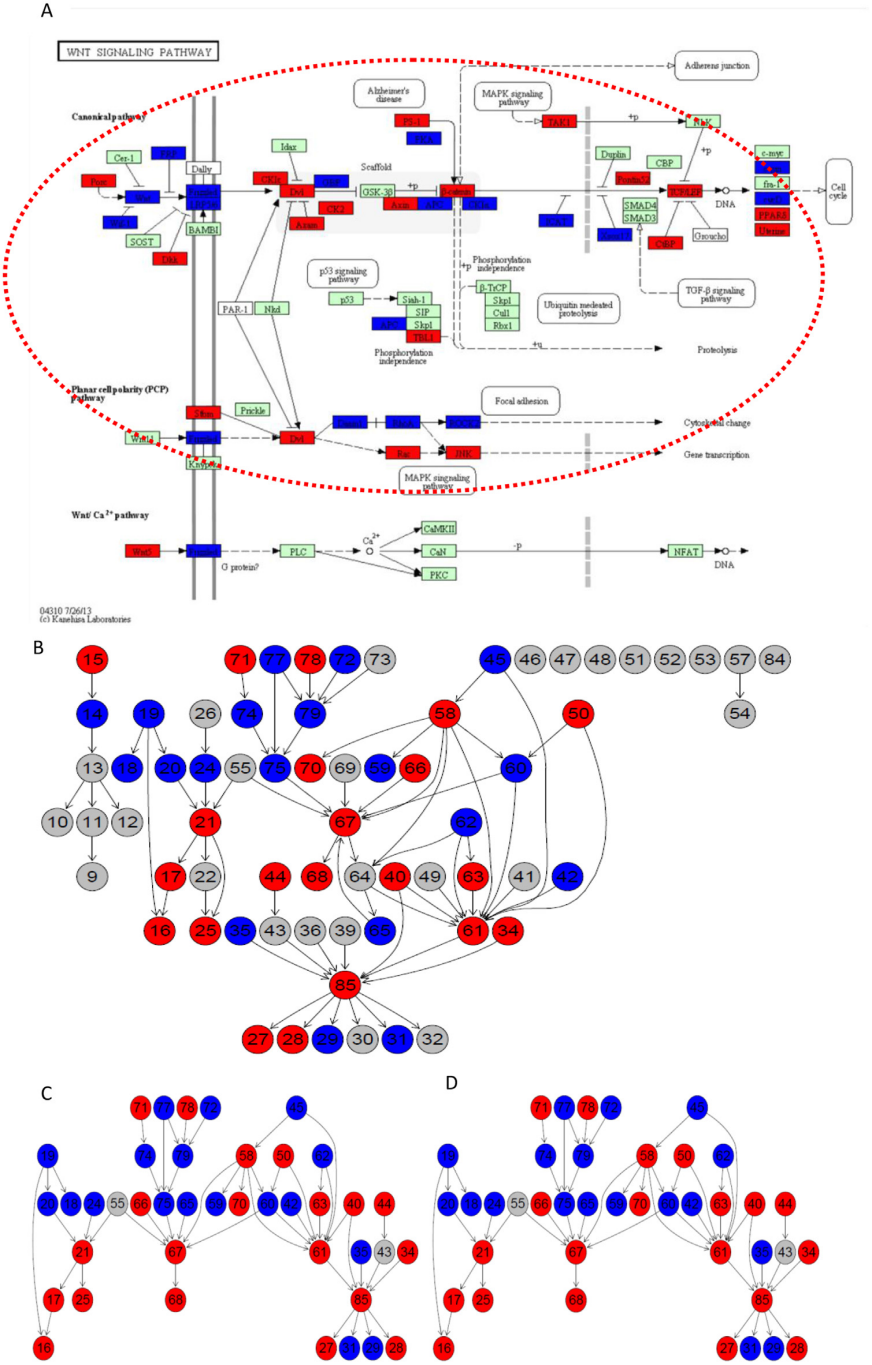
Subpathways in the Wnt signaling pathway identified by Sub-SPIA on lung cancer dataset. (A) The insulin-signal pathway with DEGs highlighted. (B) Gene network obtained by graphite package. (C) Sub Gene network corresponding to the MST for Minimal-spanning tree for *n_s_* = 4. (D) Sub Gene network corresponding to the MST for Minimal-spanning tree for *n_s_* = 2. Red indicates an up-regulated gene and blue indicates a down-regulated gene. Reprinted from http://www.kegg.jp/kegg/kegg1.html under a CC BY license, with permission from Miwako Karikomi, original copyright 2013.

The *Wnt* signaling pathway has a canonical *Wnt/β*-*catenin* cascade and two non-canonical pathways named the *Wnt/Planar* cell-polarity (*Wnt/PCP*) pathway and the *Wnt/Ca2+* pathway. Sub-SPIA identified that both the canonical *Wnt/β-catenin* cascade and the *Wnt/Planar* cell-polarity (*Wnt/PCP*) pathways are significantly enriched with DEGs relating with CRC and lung cancer. Figs 1A and 2A show the *Wnt* signaling pathway with the differentially expressed genes highlighted on the two datasets. The identified subpathways are circled by the red dashed lines for *n*_*s*_ = 4 and the blue dashed lines for *n*_*s*_ = 2. *β-catenin* was observed highly expressed in the CRC patients. According to the identified *Wnt/β-catenin* cascade, the upstream signal of *Wnt* triggers the activation of gene Frizzled and Dv1, and the downregulation of the inhibitor *GSK-3β* by *Dv1* activates the expression of *β-catenin*. Genes belonging to the *Wnt/PCP* pathway, such as *JNK* are known to be up-regulated in cancer [67]. Based on the results given in Figs 1A and 2A, we can see that gene expression of various activators and inhibitors related to *Wnt* signaling activation is consistent with the regulation flows.

Figs 1C and 2C show the gene networks corresponding to the identified subpathway for *n*_*s*_ = 4. We see that, not only do the number of DEGs differ for the two datasets, but also some DEGs exist that are highly expressed on the CRC dataset but lowly expressed on the lung dataset. this demonstrates that the *Wnt* signaling pathway may function differently in the CRC and lung cancer. For example, the downstream of the *Wnt/PCP* pathway which leads to the gene transcription in CRC is down-regulated whereas it is upregulated in lung cancer.

### Comparison with other Approaches

As mentioned in introduction, several subpathway analysis methods have been proposed to exploit the various functions of the pathway. For example, DEgraph [13] uses multivariate analysis to identify differential-expression patterns that are coherent with a given subgraph structure. The clipper approach [14] applies a Gaussian graphical model to deconstruct the pathway into smaller subgraphs (cliques). Pathiways aims to identify subpathways that have significant differences in the probability of activation of the individual stimulus-response signaling circuits. The results of applying these methods to the CRC data set and to the lung cancer dataset are included in S1 and S2 Tables, respectively.

It seems that, in comparison with sub-SPIA, the three methods mentioned above are very sensitive to the dataset. With a p-value of 0.01, Clipper, and DEgraph identified 13 and 21 significant pathways, respectively, from the CRC dataset. However, they identified many significant pathways with dubious biological meaning in the lung cancer dataset (107 and 89, respectively). Pathiways identified 10 significant pathways from the colorectal- cancer dataset but no significant pathways at all from the lung cancer data set. Therefore, in comparison, the proposed sub-SPIA generally performs more stably with both data sets.

From Tables 1 and 2, we see that some of the potential significant pathways identified by sup-SPIA were not found by these three methods, especially in the CRC data set. This result is attributed to the fact that these methods have different motivations. Although the number of significant pathways identified by the recently proposed Pathiways is relative small, but it can analyze the activity probability of a subpathway, which provides a tool with which to understand the biological mechanisms of diseases. However, these methods, including the proposed sub-SPIA method, mainly focus on identifying subpathways.

### Relationship between Identified Pathways

**Fig 3:**
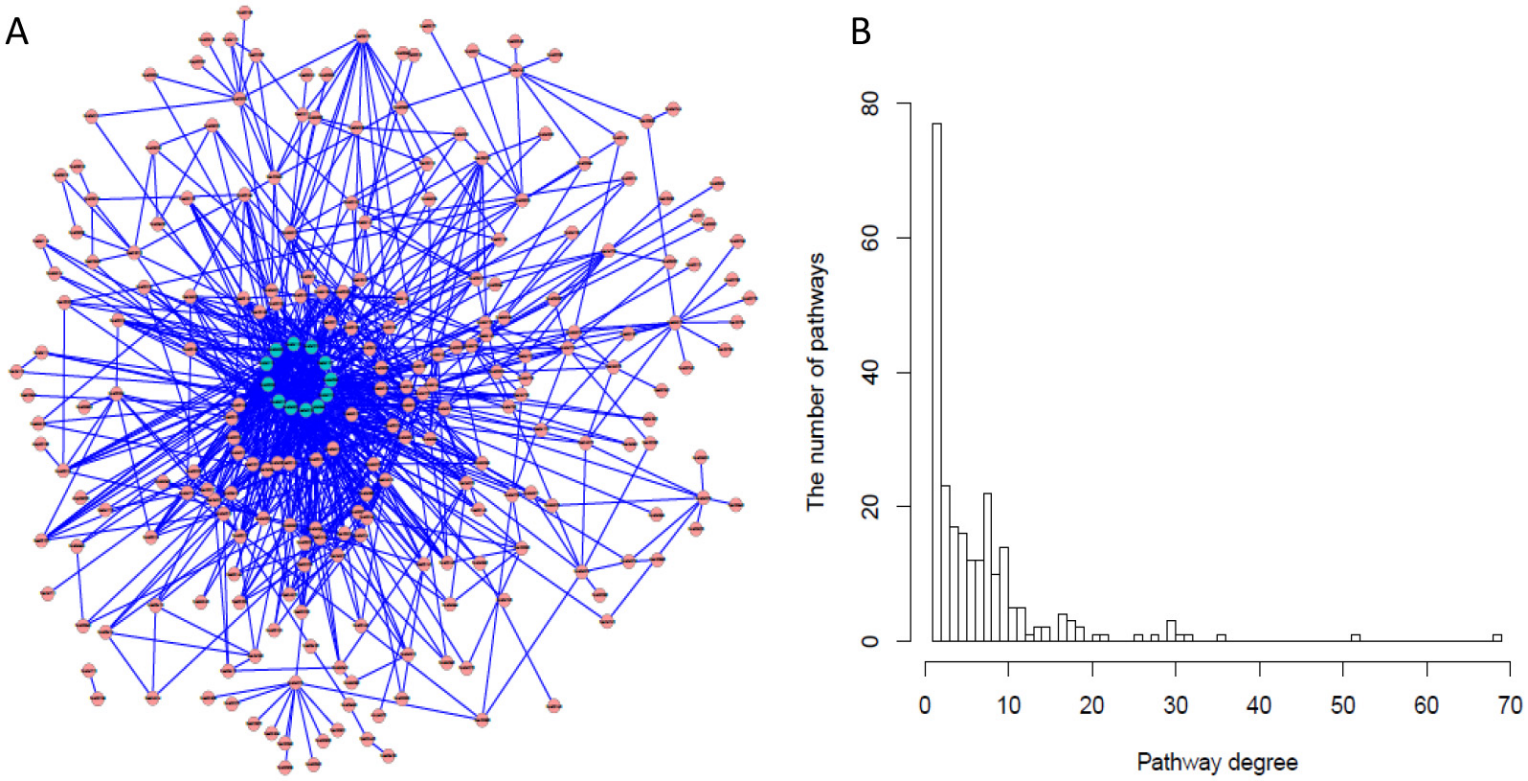
Pathway network in KEGG. (A) The connection between Pathways. The core nodes represent pathways connected with more than 20 other pathways. (B) The degree distribution of pathways in (A).

From the perspective of systems biology, the emergence and development of a cancer or disease may be due to abnormal changes in some related pathways instead of in individual pathways. Based on the connection between pathways, we construct the entire pathway network in the KEGG. Fig 3A is the pathway network that consists of 239 nodes and 818 edges. Fig 3B is its corresponding degree distribution. Each node represents a specific pathway and the edge represents the connection between nodes. Obviously, most of the pathways are sparsely connected and only a few pathways which may serve some important biological function for living systems are highly connected with other pathways. The core region in Fig 3A is the 12 pathways which connected more 20 other pathways.

To investigate the topological relationship of the significant pathways identified by each method in the entire pathway network, we extracted from Fig 3 the identified pathways and their directly connected neighbors. The average degree, clustering coefficient, and betweenness of the identified pathways are presented in Table 3. The significance of the average degree is obtained from generating 10 000 random degree distributions. The average degree of those pathways identified by sub-SPIA and Pathiways is larger than that identified by other methods, and the p-value of their average degree further indicates that they are very significant. The average betweenness of the pathways identified by sub-SPIA and Pathiways is also the largest. Both the average degree and betweenness actually reflect the hub characteristic of the identified pathways; the two observations just mentioned demonstrate that the pathways identified by sub-SPIA and Pathiways generally play important roles in the entire system.

**Table 3:** The topological characteristics of the significantly enriched pathways identified by five methods.

	Colorectal cancer				Lung cancer			
Method	Deg	p-value	Clu	Bet	Deg	p-value	Clu	Bet
**Sub-SPIA**	15.7(18)	2.0e-6	0.377	324.8	15.2(19)	1.1e-6	0.38	284.1
**SPIA**	10.6(8)	0.0933	0.33	130.4	9.4(5)	0.2379	0	94.4
**Clipper**	10.1(13)	0.0675	0	127.2	8.6(107)	0.0012	0.24	181.2
**DEGraph**	7.2(21)	0.4138	0	84.1	9.7(89)	9.6e-6	0.35	254.2
**Pathiways**	17.6(10)	9.1e-6	0	334.1	NA	NA	NA	NA

* Deg-Average Degree, Clu-Average Clustering coefficient, Bet-Average Betwenness. Number in the parentheses after Deg is the degree of the pathway in the whole pathway network. NA means there was no significant subpathways were found.

The average clustering coefficient generally reflects the degree of the closeness of the identified pathways. For both datasets, the average clustering coefficient of pathways identified by sub-SPIA is about 0.38, which is the largest obtained of the five methods. Considering the relative sparseness of the entire pathway network, this indicates that the pathways identified by sub-SPIA are more closely related than those identified by other methods. In other words, they interact closely with each other to fulfill various biological functions.

## Discussion

To identify significant pathways, we combine the subpathway analysis and the SPIA in a method we call sub-SPIA. Compared with the original SPIA, sub-SPIA dramatically improves the resolution for identifying significant pathways because subpathway analysis focuses on a local region in a pathway. From Tables 1 and 2, we see that the p-value of pathways identified by sub-SPIA is much smaller than for SPIA. Furthermore, the flexibility of the minimal-spanning tree makes it possible to capture various subpathways with complicated topologies. These two factors make sub-SPIA more sensitive than SPIA, allowing sub-SPIA to identify more potential pathways associated with specific cancers or diseases. Note that sub-SPIA misses a few pathways identified by SPIA that are related to the corresponding cancer, such as the *Epstein-Barr virus infection* [68, 69]. However, upon investigating this pathway, we find that most of the DEGs on it are scattered over the entire pathway instead of being clustered in a local region, which is why the spanning-tree algorithm fails to group them as a cluster or subpathway.

We also compared sub-SPIA with three other subpathway-analysis methods: DEgraph, Clipper, and Pathiways. Based on the number of the identified significant pathways, sub-SPIA is more stable than these three methods because they either find many dubious pathways or no significant pathways at all on the lung cancer dataset. Analyzing the relationship between the significant pathways identified by those methods further reveals that those identified by sub-SPIA not only play a more important role in the entire network but also are highly connected. Because most of these pathways are known to relate to various cancers, we may conclude that the abnormality signal propagating through them may be responsible for the specific cancer or disease.

Finally, The MST structure overcomes the disadvantage of the k-clique method, which generally results in many overlapped subpathways. For example, in the pathways identified by sub-SPIA on the two datasets with p-values of 5%, most pathways contain just one significant subpathway whereas only seven pathways contain two to five subpathways (see supplemental files). Additionally, the cross talk between pathways had been found to play an important role in cancer [70]. The flexibility of MST has the potential to find cross talk if we apply it to connected pathway networks.

## Materials and Methods

### Dataset

The first is the CRC dataset, which compares 12 CRC samples with 10 normal samples [71] by using the Affymetrix HG-U133 Plus 2.0 microarray platform (ID = GSE4107). The second dataset, which was initially analyzed by Landi *et al*. [72], is a lung cancer dataset and is publicly available at the GEO database (accession number GSE10072).This dataset contains 58 tumor samples and 49 normal samples and uses the Affymetrix Human Genome U133A Array.

### Minimal-Spanning Tree

A spanning tree is a subgraph that is a tree and connects all the vertices of the parent graph. Minimal-spanning trees (MSTs) have many applications in telecommunication and transportation-route design. As mentioned in the introduction, genes in a pathway are generally sparsely connected and the DEGs mapped in it may not be connected directly, so we search for a minimal-spanning tree that includes both the maximum number of signature nodes and the minimum number of non-signature nodes. This concept is more flexible than the k-clique concept for representing a subpathway. The Kruskal algorithm is one commonly used algorithm to find the minimal-spanning tree.

### Methods

Sub-SPIA is implemented by using the statistical programming language R and can be freely downloaded from https://github.com/eshinesimida/subpathway-analysis. The main steps to identify significantly enriched subpathways include (i) reconstruct the gene network from the signaling pathways, (ii) map the DEGs in the constructed gene network, and (iii) locate subpathways and evaluate their statistical and perturbation significance. In this work, a pathway confers significantly enriched pathways if and only if it contains at least one significant-enrichment subpathway.

### Map DEGs to graphs of pathways

We downloaded the signaling pathways from KEGG. These are directed graphs based on biochemical-reaction information in the KGML file (an XML representation of the KEGG-pathway information, see http://www.kegg.jp/kegg/xml/). The KEGG database provides one xml file for each pathway. In the KGML format, nodes in pathways often correspond to multiple gene products and compounds. Gene products can be divided into protein complexes and groups containing alternative members (gene families). We applied the graphite [73] package to reconstruct the gene network from the pathway. Next, the DEGs were mapped to the gene network.

### Locate subpathways by DEGs

DEGs within a pathway provide important signatures to locate subpathways associated with diseases of interest. Because DEGs in a subpathway are generally closely connected in the converted gene network, we first find all node sets in a pathway which include closely connected signature nodes. The main process is as follows: (i) we define a node set S = *ϕ* and add a randomly selected signature node to it. (ii) if the shortest path between a signature nodes u∈S and v∉S is less than *n*_*s*_ + 1 (parameter *n*_*s*_ is the maximum number of permitted non-signature nodes in the shortest path between two signature nodes)ns+1, then we add the non-signature nodes in their shortest path and node v to S. (iii) We repeat step (ii) until no other signature nodes can be added to S. (iv) If there exist some signature nodes in the pathway not included in the node set S, we repeat steps (i)–(iii) on them to find other node sets S′.

Obviously, genes in the node set S forms a connected sub-gene-network. However, it may include some redundant non-signature nodes that are not necessarily needed to make the signature nodes form a connected subregion. According to the definition of the MST, these redundant non-signature nodes should locate in the leaves of MST. Therefore, we first use the Kruskal algorithm to find the MSTs and then remove these non-signature nodes in the leaves of MST. We convert the subgraph of nodes in set S into an undirected weighted graph. The weight w of an edge connecting two nodes u and v is defined as

$$W=\{\begin{array}{cc} 1 & if\;u\;and\;v\;are\;signature\;nodes\\ 1+\frac{1}{k_{v}} & if\;u\;is\;a\;signature\;node\;and\;v\;is\;a\;non-signature\;node\;\\ 1+\frac{1}{k_{u}}+\frac{1}{k_{v}} & if\;u\;and\;v\;are\;non-signature\;nodes,\; \end{array}$$

where *k*_*u*_ and *k*_*v*_ are the number of signature nodes connected with nodes *u* and *v*. The Kruskal algorithm first sorts the edges in a graph according to their weight and then iteratively selects |S|-1 minimal-weighted edges that cannot form a loop with the previously selected edges at each step. After trimming these non-signature nodes in the leaves, we obtain the MST that includes the maximum number of signature nodes and the minimum number of non-signature nodes.

Flexibility can be introduced to this subpathway strategy by varying the parameter *n*_*s*_. A smaller value of *n*_*s*_ means that only those nodes meeting stricter distance similarities will be added to the corresponding subpathway, and the subpathways thus identified become smaller compared with what happens with larger values of *n*_*s*_. A smaller number of permitted non-signature nodes helps to increase the ratio of signature nodes in the located subpathway regions. In this paper, we use *n*_*s*_ = 4 to search the minimal-spanning tree based on the DEGs.

In Figs 1 and 2, we show the subpathways obtained in the Wnt signaling pathway from the CRC and lung cancer datasets by Sub-SPIA. Figs 1A and 2A show the Wnt-signal pathway with the differentially expressed genes highlighted. Figs 1B and 2B show the gene network obtained by the graphite package. Figs 1C and 2C show the subnetwork corresponding to the MST obtained by *n*_*s*_ = 4, and Figs 1D and 2D show the subnetwork corresponding to the MST obtained by *n*_*s*_ = 2. The subpathway circled by the red dashed lines in Figs 1A and 2A correspond to the subnetwork in Figs 1C and 2C. The subpathway circled by the blue dashed lines in Figs 1A and 2A correspond to the subnetwork in Figs 1D and 2D.

**Fig 4:**
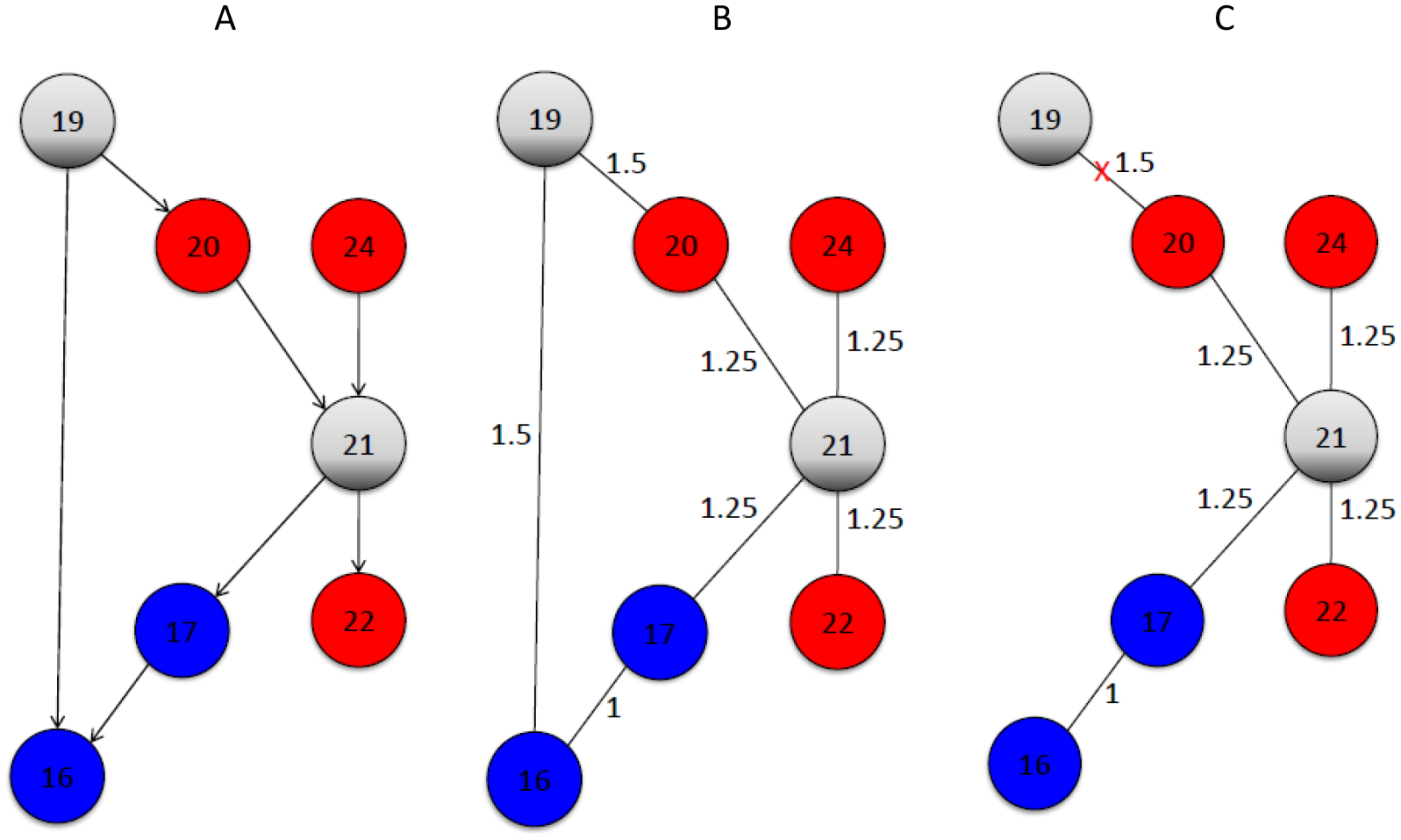
An example of the MST. (A) A sub gene network extracted from Fig 1B for *n*_*s*_ = 2. (B) The converted undirected weighted graph. (C) The resulted MST.

We now give a brief description of the MST by referring to the node set in Fig 1B. Assuming *n*_*s*_ = 2 and starting from signature node 16, we reach the node set S = {16,17,19,20,21,22,24}, which includes the two non-signature nodes 19 and 21. Fig 4A shows the sub-gene network of the nodes in Fig 1B. Fig 4B shows the converted undirected weighted graph from it. Fig 3C shows the MST obtained from the Kruskal algorithm. The final MST is obtained by removing leaf node 19, because it is a non-signature node.

### The statistical significance of subpathways

We used the hypergeometric test and abnormal perturbation to calculate the statistical significance of each subpathway. This process contains two types of evidence: the overrepresentation of DEGs and the abnormal perturbation in a given subpathway. The first probability *P*_*NDE*_ = *P*(*X*≥*N*_*de*_|*H*_0_) captures the significance of the given subpathway *P*_*i*_ by an over-representation analysis of the number of DE genes (NDE) observed on the pathway.*H*_*0*_ stands for the null hypothesis where random DEGs appear on a given subpathway. From a biological perspective, this would mean that the subpathway is not relevant to the condition under study. The value of *P*_*NDE*_ represents the probability of obtaining a number of DEGs on the given subpathway that is at least as large as the observed number *N*_*de*_. The probability *P*_*NDE*_ is obtained by assuming that *NDE* follows a hypergeometric distribution. If the whole genome has a total of *m* genes of which *t* are involved in the pathway under investigation, and the set of genes submitted for analysis has a total of *n* genes of which *r* are involved in the same pathway, then the *p-value* can be calculated to evaluate enrichment significance for that pathway as follows:

$$p=1-\sum_{x=0}^{r-1}\frac{\left(\begin{array}{l} t\\ x \end{array}\right)\left(\begin{array}{l} m-t\\ n-x \end{array}\right)}{\left(\begin{array}{l} m\\ n \end{array}\right)}$$

The second probability *P*_*PERT*_ is calculated based on the amount of perturbation measured in each pathway. A gene perturbation factor is defined as:

$$PF(g_{i})=\Delta E(g_{i})+\sum_{j=1}^{n}\beta_{ij}.\frac{PF(g_{j})}{N_{ds}(g_{j})}$$

where the term Δ*E*(*g*_*i*_) represents the signed normalized measured expression change of the gene *g*_*i*_ (log fold-change if two conditions are compared). The second term in Equation is the sum of perturbation factors of the genes *g*_*j*_ directly upstream of the target gene *g*_*i*_, normalized by the number of downstream genes of each such gene *N*_*ds*_(*g*_*j*_). The absolute value of *β*_*ij*_ quantifies the strength of the interaction between genes *g*_*i*_ and *g*_*j*_. Other detailed information can be referred in Ref. [7].

The global probability value *P*_*G*_, which tests whether the subpathway is significantly perturbed by the condition being studied, combines *P*_*NDE*_ and *P*_*PERT*_ to rank the pathways. When the null hypothesis is true, the probability of observing a pair of *p-value*s whose product, *c*_*i*_ = *P*_*NDE*_(*i*)*P_*PERT*_(*i*) is at least as low as that observed for a given pathway *P*_*i*_ is

$$P_{G}=c_{i}-c_{i}\cdot \ln(c_{i})$$

When several tens of subpathways are tested simultaneously, small *P*_*G*_ values can occur by chance. Therefore, we control the FDR of the subpathway 1% by applying the commonly used FDR algorithm [74].

## Supporting Information

S1 Table Results obtained by the five methods in CRC dataset (XLS). (XLSX)

S2 Table Results obtained by the five methods in lung cancer dataset (XLS). (XLSX)
